# Body Weight and Food/Eating-Related Behaviors During the COVID-19 Pandemic or Other Traumatic or Stressful Life Events

**DOI:** 10.3390/nu16213701

**Published:** 2024-10-30

**Authors:** Marios Argyrides, Antonios Dakanalis

**Affiliations:** 1 Department of Psychology, Neapolis University Pafos, Paphos 8042, Cyprus; m.argyrides.1@nup.ac.cy; 2 Department of Mental Health, Fondazione IRCSS San Gerardo dei Tintori, Via G.B. Pergolesi 33, 20900 Monza, Italy; 3 Department of Medicine and Surgery, University of Milano Bicocca, Via Cadore 38, 20900 Monza, Italy

## 1. Introduction

The COVID-19 pandemic has been a pivotal event, reshaping many aspects of daily life and public health across the globe. As the world grapples with the ongoing repercussions of the pandemic, research has increasingly focused on how COVID-19 and its related restrictions have impacted various aspects of human behavior and health. This Special Issue includes nine significant studies exploring the pandemic’s effects on lifestyle factors, eating behaviors, and mental health. The findings presented provide a comprehensive view of how the pandemic has influenced public health and underscore the importance of adopting informed strategies to address these impacts.

## 2. The Interplay Between Lifestyle Factors and COVID-19 Susceptibility

One critical area of research has involved understanding how lifestyle factors affect susceptibility to COVID-19. In a study by Akbar et al. [1] data from the Qatar Biobank was used to explore associations between lifestyle factors and COVID-19 susceptibility. This large-scale analysis involving 10,000 participants found that certain lifestyle choices, such as smoking status and Vitamin D supplementation, were linked to varying risks of contracting COVID-19. Specifically, current and ex-smokers had lower odds of contracting the virus compared to non-smokers, while Vitamin D supplementation was associated with an 18% reduction in the likelihood of infection. Conversely, obesity and adherence to a modern dietary pattern high in saturated fats and refined carbohydrates were positively associated with increased COVID-19 risk. These findings highlight the potential role of lifestyle modifications in mitigating the risk of infectious diseases, emphasizing the importance of public health interventions that promote healthier habits.

## 3. Pandemic Restrictions and Behavioral Changes in Specific Populations

The pandemic’s restrictions have led to significant changes in individual behaviors, particularly among vulnerable populations. Al-Haifi et al. [2] focused on Kuwaiti college students, revealing that pandemic-related restrictions had notable effects on their anthropometric measurements and lifestyle behaviors. The study found that physical activity levels and dietary habits changed significantly, contributing to changes in body weight. These findings are indicative of the impact of the pandemic on young adults and the need for targeted interventions to promote healthy lifestyle choices in this population.

Another study in this Special Issue [3] examined the effects of confinement on individuals at high risk for type 2 diabetes. Findings showed that the lockdown had a detrimental impact on participants’ health-related habits, including reduced physical activity and perceived worse diet quality. However, decreased weight and BMI were also observed, and adherence to the Mediterranean diet was improved. This mixed outcome highlights the complex nature of behavioral responses to confinement and suggests that while some individuals may adopt healthier eating patterns, others may respond in more negative ways, negatively impacting their health.

## 4. The Psychological Impact of Home Confinement and Stress

In another study in this Special Issue, Aljaadi et al. [4] assessed the effects of psychological stress resulting from home confinement and pandemic restrictions on health behaviors in Saudi Arabia and found that higher stress levels were associated with irregular eating patterns and weight gain. The study also noted that stress was linked to poorer sleep quality and disrupted eating routines, further compounding the adverse effects of confinement on health. Similarly, Foroughi et al. [5] conducted a 15-year follow-up study on women in the community, revealing an increase in eating disorder symptoms during the pandemic. The disruption of normal eating patterns, coupled with reduced social support and exercise restrictions, contributed to an increased eating disorder symptomatology. These studies emphasize the importance of addressing mental health and stress-related factors to mitigate their impact on eating behaviors and overall well-being.

## 5. Social Interactions and Eating Disorders

In another study featured in this Special Issue, Meneguzzo et al. [6] investigated how virtual social inclusion or ostracism affected emotions and eating psychopathology in patients with various eating disorders. Their study revealed that social exclusion negatively impacted psychological well-being in patients with anorexia nervosa. In contrast, those with bulimia nervosa experienced more negative effects from overinclusion. Patients with binge-eating disorder showed a reduction in specific negative emotions after overinclusion. These findings highlight the complex interplay between social dynamics and eating disorders, suggesting that both social inclusion and exclusion can significantly influence eating behaviors and emotional states.

## 6. The Impact of Chronic Stress on Childhood Obesity

In another study in this Special Issue, Piatkowska-Chmiel et al. [7] explored how chronic stress related to COVID-19 affected eating behaviors and obesity risk in children and adolescents. Their study found that chronic stress contributed to unhealthy eating patterns, weight gain, and disrupted hormonal balance, including reduced leptin and increased ghrelin levels. These hormonal changes, along with stress-induced behavioral changes, were linked to increased hunger and uncontrolled snacking. The study emphasizes the multifaceted nature of stress-related obesity and underscores the need for comprehensive preventive and intervention strategies to address these issues in children and adolescents.

## 7. Public Interest in Energy Labeling and Dietary Choices

Another aspect of pandemic-related behavioral change addressed by this Special Issue is the public’s interest in energy labeling on restaurant menus. Alkhaldy et al. [8] examined how the COVID-19 pandemic influenced interest in energy labeling and its impact on food choices. The study found that while there was some awareness of energy labeling during the pandemic, the level of interest did not significantly change compared to pre-pandemic times. This suggests that public engagement with energy labeling remains relatively low despite the increased focus on health. The findings point to a potential area for improvement in public health strategies, particularly in enhancing consumer education and engagement with nutritional information.

## 8. Systematic Review of Binge-Eating Disorder During the Pandemic

Lastly, Caldiroli et al. [9] conducted a systematic review to synthesize the impact of the COVID-19 pandemic on binge-eating disorder (BED). Their review found an increase in new BED diagnoses and in symptoms among individuals with pre-existing BED. The study highlights the vulnerability of BED patients to pandemic-related stressors and social isolation, suggesting a need for continued monitoring and targeted interventions. The review also notes the limitations of the existing literature, including the variability in study quality and heterogeneity in pandemic phases and populations.

## 9. Conclusions: A Call for Holistic Public Health Approaches

The findings presented in this Special Issue offer a better understanding of the diverse impact of the COVID-19 pandemic on lifestyle, eating behaviors, and overall health. From the effects of lifestyle factors on COVID-19 susceptibility to the psychological and behavioral changes resulting from pandemic restrictions, this Special Issue provides valuable insights into the challenges faced by different populations. The findings highlight the importance of adopting a holistic approach to public health that addresses both physical and mental health, particularly in situations of prolonged stress and social disruption.

As society continues to navigate the aftermath of the pandemic, these insights will be crucial in shaping effective public health strategies and interventions. By understanding the complex interplay between lifestyle factors, mental health, and eating behaviors, we can better address the needs of vulnerable populations and promote healthier behaviors in a post-pandemic world.
